# First person – Andrea R. López-Pastor and Jorge Infante-Menéndez

**DOI:** 10.1242/dmm.049405

**Published:** 2021-12-24

## Abstract

First Person is a series of interviews with the first authors of a selection of papers published in Disease Models & Mechanisms, helping early-career researchers promote themselves alongside their papers. Andrea R. López-Pastor and Jorge Infante-Menéndez are co-first authors on ‘ Concerted regulation of non-alcoholic fatty liver disease progression by microRNAs in apolipoprotein E-deficient mice’, published in DMM. Andrea is a PhD student in the lab of Óscar Escribano and Almudena Gómez-Hernández at Complutense University of Madrid, Madrid, Spain, investigating molecular mechanisms by which microRNAs are involved in the progression of non-alcoholic fatty liver disease. Jorge is a research assistant in the same lab, investigating translational approaches to microRNA dysregulation in non-alcoholic fatty liver disease progression.


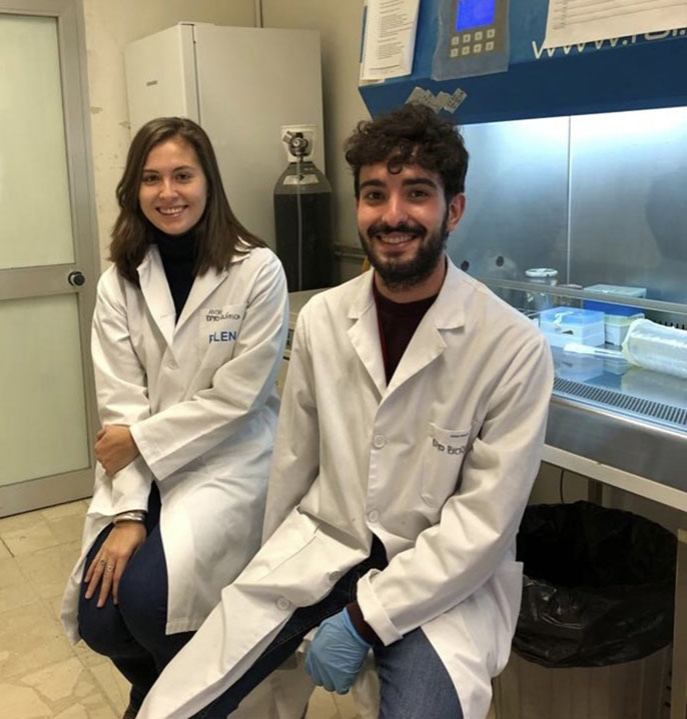


**Andrea R. López-Pastor (left) and Jorge Infante-Menéndez (right)**

**How would you explain the main findings of your paper to non-scientific family and friends?**

Obesity and overweight are characterized by an abnormal or excessive accumulation of fat, which represents a threat to health. This epidemic is a risk factor highly associated with the development of other diseases such as type 2 diabetes and cardiovascular diseases. Among these diseases, we want to highlight the subject of the article, non-alcoholic fatty liver disease (NAFLD), which is considered the most common hepatic disorder in Western countries, and the number of patients is continuously growing. The problem is that this progressive liver degeneration is silent and may not be diagnosed in a routine test, nor is there a treatment. On the other hand, microRNAs (miRNAs), very small RNA molecules, can control the decisions the cells make. In this sense, if there is a lot of a miRNA, the cells will make less of, for instance, proteins, and vice versa. So, now what? Well, we know that when we suffer from NAFLD, protein and miRNA levels are altered in the liver, which lead to the development and progression of this disease. For that reason, further research is of great need, since knowing the miRNA mechanisms of action can serve as a tool for the diagnosis and treatment of NAFLD.

“[…] microRNAs (miRNAs), very small RNA molecules, can control the decisions the cells make.”

**What are the potential implications of these results for your field of research?**

Our study is significant to the field of metabolism and, in particular, obesity and NAFLD, highly prevalent conditions worldwide. Our findings are relevant since there is not a non-invasive diagnostic tool nor an efficient treatment for NAFLD yet, exacerbating the burden that NAFLD imposes. For that reason, we propose some miRNAs as regulators of disease development and new potential biomarkers for diagnosis. For instance, we would like to highlight miR-26b-5p as a therapeutic target, and let-7d-5p as a non-invasive biomarker.

**What are the main advantages and drawbacks of the model system you have used as it relates to the disease you are investigating?**

A great advantage of this study is that our results support previous findings proposing *Apoe* knockout mice, a traditional atherosclerosis model, as a suitable model of NAFLD. We hope that our research will encourage other NAFLD researchers to consider this model for their studies. The main drawback would be the lack of an experimental group consisting of wild-type mice fed a high-fat diet, since we think that our results could have been even clearer.

**Figure:**
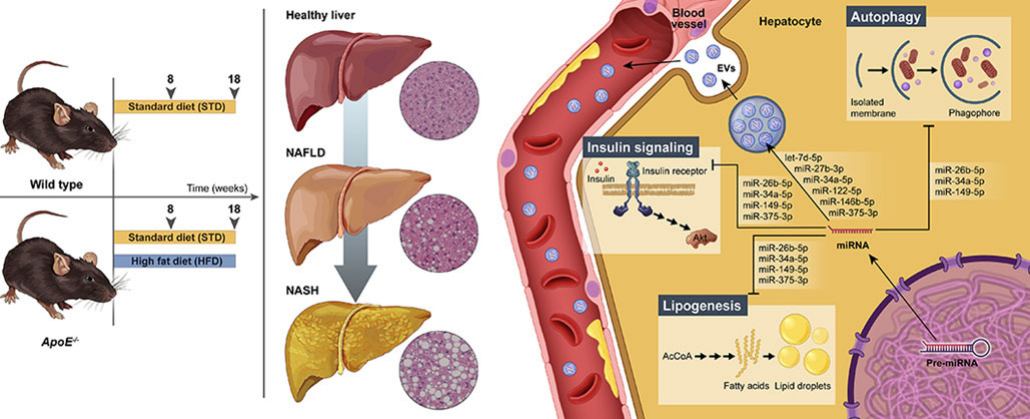
Schematic representation of the main features of NAFLD in the *Apoe^−/−^* mouse model, highlighting the noticeable histological differences between the experimental groups and the selected microRNAs as regulators of disease development, and potential biomarkers for its diagnosis.

**What has surprised you the most while conducting your research?**

**A.R.L.-P.:** I would say what has surprised me the most was how the dysregulated expression of certain miRNAs could trigger NAFLD development as well as control their target levels. I mean that these tiny molecules are able to manage the function of our tissues, which makes them very powerful.

**J.I.-M.:** For me, the most surprising finding was the abrupt changes in miRNA expression that we observed between the distinct stages of NAFLD progression, since the changes we observed in earlier phases were more prominent in the most severe stages. This discovery made me realize how crucial these small molecules are for the maintenance of proper cell and tissue functions.

**Describe what you think is the most significant challenge impacting your research at this time and how will this be addressed over the next 10 years?**

**A.R.L.-P.:** The main challenge is to discover a tool able to treat liver damage or a tool capable of identifying the most influential miRNAs in the liver of each NAFLD patient. In the near future, this will be addressed by finding compatible resources with humans and promoting networking between research groups that focus on the same, as well as fostering translational medicine.

**J.I.-M.:** The most challenging aspect of our research will be the translation of our findings to clinical settings. In order to develop novel therapeutic and diagnostic tools for NAFLD, especially when using molecules that exert such complex regulation, interindividual features will have to be taken into account. I think that miRNA-based strategies will pave the way towards the consolidation of precision and individualized medicine, which will be made much easier with the help of high-throughput techniques such as RNA sequencing.

**What changes do you think could improve the professional lives of early-career scientists?**

**A.R.L.-P.:** From my point of view, as I mentioned in a previous interview, one of the main concerns of early-career scientists is funding, especially here in Spain due to low accessibility and investment. For that reason, certain financial grants should be provided by governments or private associations to those PhD students who do not have a scholarship or research contract in order to thank them for their contribution to the scientific community. In addition, due to the impact of the COVID-19 pandemic, it is crucial to increase travel grants for PhD scientists to improve their research and foster training courses to complete their CV.

**J.I.-M.:** Aside from promoting the access of young investigators to grants, scholarships and contracts, I think that dissemination programs could help university students to get familiarized not only with research topics and techniques, but also with the workflow of a laboratory, as well as develop critical scientific thinking. This would be highly helpful for young investigators, since it would lead to the production of high-quality contributions that could help them achieve successful careers in research.

“[…] one of the main concerns of early-career scientists is funding […]”

**What's next for you?**

In terms of this study, the next step is to unravel similar mechanisms by which miRNAs are involved in the progression of human fatty liver patients.

**A.R.L.-P.:** For me personally, the next goal is to finish my PhD thesis within the next few months.

**J.I.-M.:** Personally, my long-term goals include obtaining my PhD and continuing to decipher the progression mechanisms of metabolic diseases, particularly NAFLD.
